# Delta-shaped gastroduodenostomy using a robotic stapler in reduced-port totally robotic gastrectomy: its safety and efficiency compared with conventional anastomosis techniques

**DOI:** 10.1038/s41598-020-71807-z

**Published:** 2020-09-07

**Authors:** Ji Su Kim, Hemant Batajoo, Taeil Son, Seohee Choi, Won Jun Seo, Minah Cho, Yoo Min Kim, Joong Ho Lee, Hyoung-Il Kim, Woo Jin Hyung

**Affiliations:** 1 Department of Surgery, Yonsei University College of Medicine, 50 Yonsei-ro Seodaemun-gu, Seoul, 03722 Republic of Korea; 2 Department of Surgery, Nepal Cancer Hospital and Research Center, Lalitpur, Nepal; 3 Gastric Cancer Center, Yonsei Cancer Hospital, Yonsei University Health System, Seoul, Republic of Korea; 4 Department of Surgery, Korea University College of Medicine, Seoul, Republic of Korea; 5 Department of Surgery, Yongin Severance Hospital, Yonsei University College of Medicine, Gyeongi, Republic of Korea

**Keywords:** Surgical oncology, Gastric cancer

## Abstract

To investigate the safety and efficiency of using robotic staplers for intracorporeal gastroduodenostomy in reduced-port robotic gastrectomy for gastric adenocarcinoma. We retrospectively reviewed patients who underwent totally robotic and laparoscopic gastrectomy with intracorporeal gastroduodenostomy. Gastroduodenostomy using the ENDOWRIST robotic stapler (RR) was compared to that using an endolinear stapler during robotic gastrectomy (RE) and to that using an endolinear stapler during laparoscopic gastrectomy (LE). A total of 296 patients underwent gastroduodenostomy: 58, 28, and 210 patients with RR, RE, and LE, respectively. There were no conversions to other methods, and all robotic stapling procedures were performed on the console without receiving additional assistance from a bedside surgeon during RR. Comparing the operative outcomes of RR with those of RE and LE, respectively, we noted similar postoperative short-term outcomes. There were no major complications, including anastomosis-related complications, during the postoperative period after RR. The median reconstruction time during RR was 8 min and 45 s, which was similar to that during RE (8 min, 5 s [*P* > 0.9999]), but longer than that during LE (6 min, 30 s [*P* < 0.0001]). Intracorporeal gastroduodenostomy using the robotic stapler during robotic gastrectomy could be safely and feasibly performed on the console without the assistance of assistant, bedside surgeons.

## Introduction

With laparoscopic gastrectomy proving to be better in treating early stage gastric cancer and with it becoming regarded as a standard alternative treatment of gastric cancer^1–3^, minimally invasive surgical approaches for gastric cancer have been rapidly adopted in East Asia^4–8^. One recent advance in minimally invasive surgery for gastric cancer is totally minimally invasive gastrectomy, in which all procedures, including anastomosis, are performed intracorporeally. However, during totally minimally invasive gastrectomy, restoration of intestinal continuity is technically demanding and requires extensive experience among the surgical team^9–11^. When performed intracorporeally, gastroduodenostomy, a common anastomosis procedure performed after distal subtotal gastrectomy, is thought to be more difficult than other anastomosis methods for most surgeons^12^. Accordingly, we sought to develop a method for intracorporeal gastroduodenostomy that is safe and can be performed with less effort during gastric cancer surgery, including robotic gastrectomy^9^.

In robotic surgery, an assistant surgeon who stands over the patient is needed to control laparoscopic endolinear staplers^11^. If the stapling in gastroduodenostomy is not performed by a skilled assistant, the security of the anastomosis may be compromised. Furthermore, since the surgeon is positioned at the console to control the robot arms, it can be difficult to ascertain the movement of the assistant surgeon. Therefore, the role of and cooperation with the assistant surgeon for anastomosis are crucial.

Gastroduodenostomy can be much more demanding when gastrectomy is performed with reduced-port surgery, which affords limited access and restricted movement of the surgical instruments. Thus, gastroduodenostomy during reduced-port surgery is rarely performed and reported^9^. Recently, a robot-controlled stapler (ENDOWRIST Stapler, Intuitive Surgical, Sunnyvale, CA, US) was developed for robotic surgical systems to help facilitate intracorporeal anastomosis by the surgeon on the console. The aim of this study was to investigate the safety and efficacy of a robotic stapling technique for delta-shaped gastroduodenostomy in reduced-port totally robotic gastrectomy. We compared perioperative outcomes, including the reconstruction time of intracorporeal gastroduodenostomy, with this new technology to those with intracorporeal gastroduodenostomy using laparoscopic endolinear staplers during totally robotic or laparoscopic gastrectomy.

## Results

### Preoperative clinicopathologic characteristics

The preoperative characteristics of the 296 patients are summarized in Table 1. Among these patients, 58, 28, and 210 patients were treated with RR, RE, and LE, respectively. Groups RR and RE showed no significant differences in age, sex, American Society of Anesthesiologists (ASA) classification, body mass index (BMI), cT and cN classification, and tumor location. There were also no significant differences in preoperative characteristics between groups RR and LE, except for median age (*P* = 0.0021).

**Table 1:** Preoperative clinicopathologic characteristics of the patients in Groups RR, RE, and LE.

Variables	Group RR (*n* = 58)	Group RE (*n* = 28)	*P* value	Group LE (*n* = 210)	*P* value
Age (years)	55 (47.0–61.0)	51 (44.5–55.5)	> 0.9999	60 (53.0–71.0)	0.0021
**Sex**			> 0.9999		> 0.9999
F	24 (41.4%)	14 (50.0%)		100 (47.6%)	
M	34 (58.6%)	14 (50.0%)		110 (52.4%)	
**ASA class**			> 0.9999		0.3888
1	10 (17.2%)	6 (21.4%)		36 (17.1%)	
2	40 (69.0%)	16 (57.1%)		114 (54.3%)	
3	8 (13.8%)	6 (21.4%)		56 (26.7%)	
4	0 (0.0%)	0 (0.0%)		4 (1.9%)	
BMI (kg/m^2^)	24.2 (21.8–25.4)	23.8 (21.3–25.3)	> 0.9999	23.5 (21.1–25.6)	0.7104
**cT classification**			> 0.9999		0.4845
T1	47 (81.0%)	21 (75.0%)		183 (87.1%)	
T2	9 (15.5%)	6 (21.4%)		20 (9.5%)	
T3	1 (1.7%)	1 (3.6%)		7 (3.3%)	
T4a	1 (1.7%)	0 (0.0%)		0 (0.0%)	
**cN classification**			> 0.9999		0.3051
0	52 (89.7%)	26 (92.9%)		201 (95.7%)	
1	6 (10.3%)	2 (7.1%)		9 (4.3%)	
**Tumor location**			0.452		0.678
Lower	36 (62.1%)	15 (53.6%)		124 (59.0%)	
Middle	22 (37.9%)	13 (46.4%)		86 (41.0%)	

Variables are shown as medians [interquartile range Q1–Q3] or n (%).

*RR* robotic distal gastrectomy with gastroduodenostomy using the ENDOWRIST stapler, *RE* robotic distal gastrectomy with gastroduodenostomy using a laparoscopic endolinear stapler, *LE* laparoscopic distal gastrectomy with gastroduodenostomy using a laparoscopic endolinear stapler, *ASA class* American Society of Anesthesiologists classification, *BMI* body mass index, *LN* lymph node, *TNM stage* tumor node metastasis stage.

### *Perioperative outcomes* (Table 2)

**Table 2:** Perioperative outcomes for the three groups.

Variables	Group RR (*n* = 58)	Group RE (*n* = 28)	*P* value	Group LE (*n* = 210)	*P* value
LN extent			> 0.9999		0.3249
D1+	46 (79.3%)	21 (75.0%)		184 (87.6%)	
D2	12 (20.7%)	7 (25.0%)		26 (12.4%)	
Operation time (min)	174.5 (161.0–201.0)	207 (175.5–222.5)	0.0174	148.5 (125.0–187.0)	< 0.0001
Estimated blood loss (ml)	20.5 (15.0–30.0)	29 (17.0–85.5)	0.1059	50 (30.0–90.0)	< 0.0001
Proximal margin (mm)	39 (25.0–57.0)	46.5 (20.0–60.0)	> 0.9999	42 (30.0–63.0)	0.6801
Proximal margin involvement	1 (1.7%)	0 (0%)	> 0.9999	0 (0%)	0.6402
Distal margin (mm)	44 (24.0–70.0)	44.5 (18.0–75.0)	> 0.9999	51.5 (33.0–80.0)	0.2622
Distal margin involvement	0 (0%)	0 (0%)	–	0 (0%)	–
No. of metastatic LNs	0 (0–0)	0 (0–0)	0.9735	0 (0–0)	> 0.9999
No. of retrieved LNs	42.5 (35.0–54.0)	48.5 (35.5–69.5)	0.9402	42 (33.0–55.0)	> 0.9999
pTNM stage 8th			> 0.9999		> 0.9999
IA	46 (79.3%)	19 (67.9%)		174 (82.9%)	
IB	4 (6.9%)	6 (21.4%)		13 (6.2%)	
IIA	2 (3.4%)	1 (3.6%)		14 (6.7%)	
IIB	2 (3.4%)	1 (3.6%)		3 (1.4%)	
IIIA	3 (5.2%)	1 (3.6%)		3 (1.4%)	
IIIB	1 (1.7%)	0 (0%)		3 (1.4%)	
Gas passing (POD)	3 (3–4)	3 (3–3)	0.2241	3 (3–4)	> 0.9999
Liquid diet (POD)	3 (3–3)	3 (3–3)	> 0.9999	3 (3–3)	> 0.9999
Soft diet (POD)	4 (4–4)	4 (4–4)	> 0.9999	4 (4–4)	> 0.9999
Hospital stay (POD)	5 (5–6)	5 (5–6)	> 0.9999	5 (5–6)	0.3396
Major complication					> 0.9999
Yes	0 (0%)	0 (0%)		2 (1.0%)	
Anastomosis stenosis	0	0		1	
Pneumonia	0	0		1	
Minor complication	11 (19%)	5 (17.9%)	> 0.9999	39 (18.6%)	> 0.9999
Gastric stasis	1 (1.7%)	0 (0.0%)		4 (1.9%)	
Others	10 (17.2%)	5 (17.9%)		35 (16.7%)	

Variables are shown as medians [interquartile range Q1–Q3] or n (%).

*RR* robotic distal gastrectomy with gastroduodenostomy using the ENDOWRIST stapler, *RE* robotic distal gastrectomy with gastroduodenostomy using a laparoscopic endolinear stapler, *LE* laparoscopic distal gastrectomy with gastroduodenostomy using a laparoscopic endolinear stapler, *POD* postoperative day, *LN* lymph node, *TNM stage* tumor node metastasis stage.

No incomplete fires of the robotic stapler in group RR occurred during the surgical procedures. The median operation time of RR was 174.5 min and was significantly shorter than that of RE, which was 207 min (*P* = 0.0174), but longer than that of LE (148.5 min [*P* < 0.0001]). Estimated blood loss totaled 20.5 ml in group RR, which was similar to that in group RE (29 ml, *P* = 0.1059), but significantly less than that in group LE (50 ml, *P* < 0.0001). Postoperative hospital stay did not differ between groups RR and RE (*P* > 0.9999) and between groups RR and LE (*P* = 0.3396). An in-hospital major complication (grade III or more severe) was noted only in two patients in group LE: one was postoperative pneumonia that required intensive care, and the other was anastomosis stenosis that required endoscopic balloon dilatation. There were no differences in the incidences of minor complications between the groups (19% in group RR vs. 17.9% in group RE [*P* > 0.9999] and vs. 18.6% in group LE [*P* > 0.9999]). Anastomosis-related complications (gastric stasis) were noted in 1 (1.8%) patient in group RR and 4 (1.9%) patients in group LE; all were managed conservatively.

Patients were followed at a median of 34 months postoperatively. Postoperative upper endoscopy was performed at around 12 months except in nine patients who were lost to follow-up, revealed no anastomosis-related complications, such as stricture or narrowing, among all studied patients. The incidence of food stasis at the time of upper endoscopy, which would represent delayed gastric emptying, was similar among comparisons.

### Reconstruction times among the three groups

**Figure 1:**
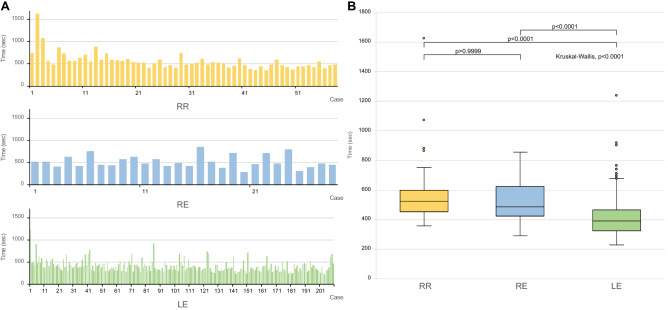
Comparison of total reconstruction times. (**A**) Bar plots indicate the reconstruction times for each case in the study groups. (**B**) Box plots depicting the comparison of total reconstruction times for the study groups.

**Figure 2:**
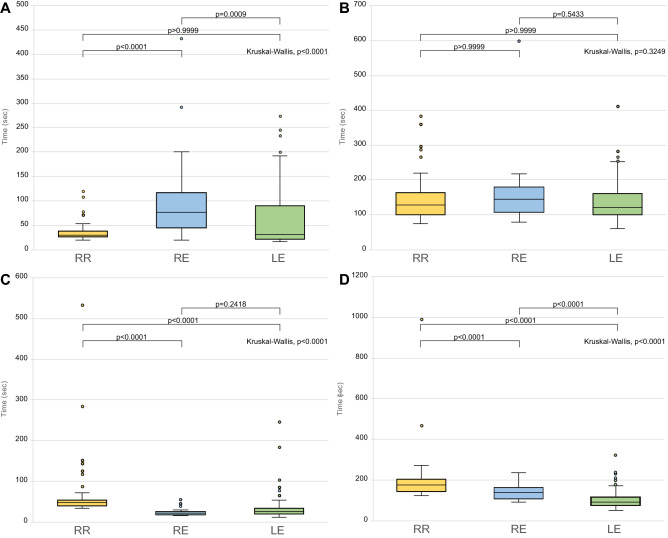
Comparison of surgical steps during intracorporeal delta-shaped gastroduodenostomy. (**A**) Time for creation of a hole in the remnant stomach and the duodenum. (**B**) Time for creation of a common channel between the stomach and the duodenum. (**C**) Time for preparing for a second stapler. (**D**) Time for closing the entry hole using two firings of the staplers.

Reconstruction times are depicted in Table 3. We noted no significant difference in median reconstruction times between group RR and group RE (8 min, 45 s vs. 8 min, 5 s, respectively, *P* > 0.9999); however, group RR had a longer reconstruction time than group LE (6 min, 30 s, *P* < 0.0001) (Fig. 1). The time required to create entry holes in the duodenum and the stomach in group RR (29 s) was shorter than that in group RE (1 min, 16 s, *P* < 0.0001), but similar to that in group LE (32 s, *P* > 0.9999). There was no significant difference in the median times for common channel creation between groups RR and RE (2 min, 8 s vs. 2 min 24 s, respectively, *P* > 0.9999) and between RR an LE (2 min, 2 s, *P* > 0.9999). The time taken to prepare the second stapler in group RR (47 s) was significantly longer than that in group RE (21 s, *P* < 0.0001) and in group LE (25 s, *P* < 0.0001). Similarly, closure of the entry hole took longer in group RR (2 min, 54 s) than in group RE (2 min, 18 s, *P* < 0.0001) and in group LE (1 min, 32 s, *P* < 0.0001) (Fig. 2).

**Table 3:** Comparison of the reconstruction time for each method.

Variables	Group RR (*n* = 58)	Group RE (*n* = 28)	*P* value	Group LE (*n* = 210)	*P* value
Total reconstruction time					< 0.0001
	8:45 [7:37–9:59]	8:05 [7:05–10:06]	> 0.9999	6:30 [5:22–7:44]	< 0.0001
Creation of the entry holes					< 0.0001
	0:29 [0:26–0:37]	1:16 [0:46–1:54]	< 0.0001	0:32 [0:21–1:30]	> 0.9999
Creation of the common channel					0.3249
	2:08 [1:41–2:39]	2:24 [1:47–3:00]	> 0.9999	2:02 [1:40–2:41]	> 0.9999
Preparation of the second stapler					< 0.0001
	0:47 [0:40–0:53]	0:21 [0:18–0:25]	< 0.0001	0:25 [0:19–0:34]	< 0.0001
Closure of the entry hole					< 0.0001
	2:54 [2:25–3:21]	2:18 [1:46–2:41]	< 0.0001	1:32 [1:16–1:55]	< 0.0001

All values are medians (min:s) [interquartile range Q1–Q3]. The Kruskal–Wallis test was conducted to identify differences among the three groups. Post-hoc analysis was performed applying the Bonferroni method.

*RR* robotic distal gastrectomy with gastroduodenostomy using the ENDOWRIST stapler, *RE* robotic distal gastrectomy with gastroduodenostomy using a laparoscopic endolinear stapler, *LE* laparoscopic distal gastrectomy with gastroduodenostomy using a laparoscopic endolinear stapler.

## Discussion

In this study, we demonstrated that robotic ENDOWRIST staplers, which can be fully controlled by the surgeon at the console, can be safely and efficiently applied for performing intracorporeal delta-shaped gastroduodenostomy, which is regarded as a technically demanding procedure, during robotic distal gastrectomy without the need for assistance from an assistant, bedside surgeon. In this study, although the reconstruction time for the procedure was longer than that for gastroduodenostomy during conventional laparoscopic gastrectomy, which was performed by the operating surgeon, it was similar to that of another anastomosis technique during robotic surgery in which laparoscopic endolinear staplers were manipulated by assistant surgeons who are skilled in this procedure. When we divided and evaluated reconstruction time into specific steps, we found that the robotic stapler group had similar or longer times for each step than the other endolinear stapler groups. Remarkably, however, the times to create a common channel between the duodenum and the stomach, which we consider the most challenging part of intracorporeal gastroduodenostomy, were similar. Moreover, the median reconstruction time, which was recorded at 8 min and 45 s, was comparable and even faster than the previously reported reconstruction time for laparoscopic or robotic delta-shaped anastomosis^11,13^. This is probably due to the advantages of the robotic stapler, as well as our anastomosis technique, which obviated the need for stay sutures to retract the intestines.

Gastroduodenostomy is the most commonly performed method of anastomosis after distal subtotal gastrectomy. More than 60% of patients enrolled in multicenter randomized clinical trials that have allowed any type of anastomosis have undergone gastroduodenostomy after distal gastrectomy^5,8^. Delta-shaped gastroduodenostomy with laparoscopic endolinear stapler was first introduced by Kanaya and his colleagues, and applications for intracorporeal anastomosis have continued to grow in minimally invasive gastrectomy, especially in Korea and Japan^12,14,15^. However, it is still regarded as technically challenging because tension on the intestines should be adequately controlled by retracting the duodenum and the stomach from multiple directions. As experience and understanding of the procedure by both the operating surgeon and the surgical team are crucial to successfully completing this technique, it is not commonly performed when a new gastrectomy procedure is introduced for the treatment of gastric cancer. Nevertheless, despite reporting a reluctance with performing intracorporeal gastroduodenostomy during reduced-port or single incision gastrectomy^9^, we have demonstrated that a novel intracorporeal delta-shaped gastroduodenostomy technique could be successfully carried out, even during reduced-port robotic gastrectomy with SINGLE-SITE and two additional ports^9,16^.

Having the ability to perform anastomosis intracorporeally by the surgeon in a safe and efficient manner on the console is desirable. During robotic gastrectomy, the surgeon is often unable to ascertain the movement of their bedside assistants, making it difficult to restore intestinal continuity using staplers. For this reason, some surgeons have attempted to achieve anastomosis with a robot-sewn technique from the console or with a hybrid technique wherein the operating surgeon scrubs and performs laparoscopic anastomosis at the bedside^17,18^. In addition, it is much more challenging to perform anastomosis during reduced-port surgery, which has constricted access and a limited range of motion for instruments. To solve these problems, we have implemented the use of a robotic stapler. With the robotic stapler, surgeons can take advantage of dual-side, wristed articulation, which provides wider side-to-side and up and down articulation than any other current laparoscopic endolinear stapler. This dual direction and wider articulation of the stapler provides better movement and positioning of staplers in the abdominal cavity, regardless of the trocar position for the stapler. The robotic stapler also provides feedback on tissue thickness prior to firing and on whether the jaws are suitably closed on the tissue for formation of a good staple line.

This study, as far as we know, is the first to investigate the efficacy and safety of the use of a robotic stapler for gastroduodenostomy by evaluating reconstruction time in detail during robotic gastrectomy for gastric cancer. Although this study was conducted retrospectively, the times for reconstruction were accurately measured by reviewing video recordings of operations. In addition, only 11% (37/333) of the indicated patients were excluded, primarily because of different stapling devices or usages. All studied patients underwent the exact same anastomosis technique by an experienced surgical team at a high-volume center. However, there are still some limitations to the study. First is that the three different techniques reflect different stages of the learning curve and the comparison between the two robotic surgery groups was not entirely adequate in terms of the period of the study, because the RE group was mainly treated during our initial experience with robotic surgery. For this reason, operation times in group RE significantly differed from those in group RR, operations which were performed more recently. Secondly, cost analysis was not conducted. We routinely used seven firings of the staplers for anastomosis, including gastric and duodenal division, in each case. The cost of seven firings of the robotic stapler is much more expensive than that of seven firings of the laparoscopic endolinear staplers used at the time of this study by approximately 1,400 USD. Spending that cost to reduce dependency on assistance from an assistant surgeon during anastomosis could be controversial in other countries, as well as in Korea. Currently, we are performing surgery with the combined use of robotic and laparoscopic endolinear staplers to reduce costs in light of the results of the current study. Lastly, there is a selection bias mainly on the patient’s preference and the reasoning to choose one approach over the other which has long been pointed out in this field.

In conclusion, intracorporeal delta-shaped gastroduodenostomy using the robotic stapler during reduced-port totally robotic gastrectomy could be safely and effectively performed on the robotic console without the need for a bedside assistant. Reconstruction times were acceptable and similar to those of robotic anastomosis assisted by experienced bedside assistants. Despite the positive results, an improved form of the robotic stapler that functions faster and is less expensive is desirable.

## Methods

### Patients

The medical records and operation videos of patients who underwent minimally invasive surgery for primary gastric adenocarcinoma from September 2015 to September 2018 in the Department of Surgery, Yonsei University College of Medicine in Seoul, Korea were retrospectively evaluated. All cases were treated by a single surgeon with either DA VINCI robotic or laparoscopic distal subtotal gastrectomy with lymph node dissection based on the standard guidelines^19,20^. The indications for minimally invasive surgery were early gastric cancer not indicated for endoscopic procedure up to advanced gastric cancer involving the serosa layer of the stomach. The patients were involved in the decision-making process and were allowed to choose either robotic or laparoscopic gastrectomy. Written informed consent was obtained from all patients. The study protocol was approved by the institutional review board of Severance Hospital, Seoul, Korea (4-2017-0938).

**Figure 3:**
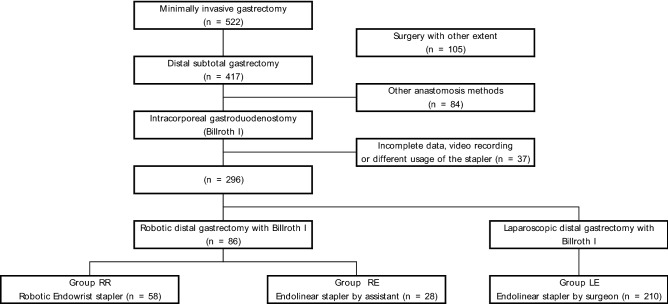
Study design.

The patient inclusion criteria are summarized in Fig. 3. During the study period, 522 patients underwent robotic or laparoscopic gastrectomy for primary gastric cancer. Patients (n = 105) who underwent surgery to other extents, for instance, proximal, total, and completion total gastrectomy for remnant stomach cancer, were excluded. Among 417 patients who underwent distal gastrectomy, 84 patients were excluded due to other types of anastomosis, for instance, gastrojejunostomy (Billroth II) or Roux-en-Y gastrojejunostomy. Thirty-seven patients with incomplete information on perioperative data, no video recording, or different usage of the stapler were finally excluded. Of all 296 patients who met the inclusion criteria, 58 patients who underwent gastroduodenostomy using the ENDOWRIST robotic stapler during robotic gastrectomy (Group RR), 28 patients using an endolinear stapler controlled by a bedside assistant surgeon during robotic gastrectomy (Group RE), and 210 patients using an endolinear stapler controlled by the operating surgeon during laparoscopic gastrectomy (Group LE) were enrolled and analyzed. All robotic procedures in group RR were performed with the SINGLE-SITE plus two port system as described in the literature^9,16^ and the robotic procedures in group RE were performed using either a conventional five-port or reduced-port system, primarily during the initial experience with robotic surgery. In the laparoscopic procedures in Group LE, five ports were routinely used. All methods were carried out in accordance with relevant guidelines and regulations.

### Surgical technique

#### Robotic gastrectomy

Under general anesthesia, the patient was placed in the reverse Trendelenburg position. In robotic surgery, reduced-port distal subtotal gastrectomy was performed using both the DA VINCI SI and XI Surgical Systems (Intuitive Surgical, Sunnyvale, CA, USA) as described in our previous report^16^. The reduced-port approach utilized a SINGLE-SITE port and two additional straight cannulas. In our initial experience with robotic gastrectomy, a conventional robotic approach was performed using five ports, as well described in the literature^21,22^. To achieve delta-shaped gastroduodenostomy intracorporeally, the duodenum should be transected in a posteroanterior direction, which is rotated from the usual direction, in order to maintain favorable blood supply to the anastomotic line in the same manner as that during laparoscopic intracorporeal gastroduodenostomy^12^. After adequate lymph node dissection based on guidelines, delta-shaped anastomosis was completed as described previously^9^. Briefly, the first step was to create small entry holes through which to introduce the staplers: one at the mesenteric side of the duodenal stump and one at the tip of the greater curvature of the remnant stomach. Then, suction was inserted from the patient’s left side, and all gastric contents were sucked out.

Next, the jaws of the 45-mm robotic ENDOWRIST stapler (Intuitive Surgical, Sunnyvale, CA, USA) in RR or a 45-mm laparoscopic flexible endolinear stapler (ECHELON FLEX Ethicon Endo-Surgery, Cincinnati, OH, USA) in RE were inserted into the holes to form a side-to-side gastroduodenostomy between the posterior wall of the remnant stomach and the duodenum. The entry hole for the stapler was closed by two firings of the 45-mm robotic or endolinear stapler. The robotic stapler obviated the need for support from an assistant surgeon during anastomosis; however, additional time was required for installation, calibration, clamping, firing, and unclamping of the stapler. Meanwhile, the laparoscopic endolinear stapler was manipulated by a bedside assistant surgeon familiar with robotic and laparoscopic delta-shaped gastroduodenostomy formation.

#### Laparoscopic gastrectomy

The laparoscopic approach utilized five ports for all cases. A 12-mm trocar for a 30° camera was inserted below the umbilicus using an open technique. The other four trocars were then inserted: two 12-mm trocars into the right and left mid-abdomen and two 5-mm trocars into the right and left upper abdomen. The duodenum was transected in the same fashion by the surgeon with the 45-mm endolinear stapler (ECHELON FLEX), which was inserted from the 12-mm trocar in the left abdomen. All stapling procedures were manipulated by the surgeon or assistant surgeon on the patient’s left side as originally described by Kanaya et al.^12^. After creating V-shaped anastomosis, the entry hole was closed in the same manner by two firings of the 45-mm endolinear stapler.

### Measurements

#### Surgical outcomes

Patients were observed for at least 5 days after surgery. Clinicopathological characteristics and short-term surgical outcomes, including length of postoperative hospital stay, restoration of bowel function, and complications, were assessed. Postoperative complications were stratified according to Clavien–Dindo classification. All grade III or more severe complications, as well as anastomosis-related complications, were analyzed^23,24^. Patients were followed every 3 months up to 6 months after the operation and then every 6 months until 24 months after the operation. They were then followed every 6 or 12 months until 60 months. Routine follow-up upper endoscopy was performed at around 12 months and followed every 12 months after the operation. Any late-period anastomosis-related complications were checked and assessed.

#### Reconstruction time

Total operation time was defined as the time from the abdominal incision to complete closure of the port sites. Reconstruction time in the current study was recorded as the time required to make an entry hole either at the tip of the duodenum or the remnant stomach until closure of the entry hole for the stapler by firing with the last stapler, as previously defined^11^. For all cases in the current study, three 45-mm robotic or laparoscopic endolinear staplers were used to create the common channel of the gastroduodenostomy and to close the entry hole, as originally described in the literature^12^. To evaluate reconstruction time in greater detail in an attempt to better assess the efficacy of gastroduodenostomy with each stapling technique, we divided reconstruction time into five steps as follows: (1) creation of the entry holes, which started with the insertion of ultrasonic shears (HARMONIC SCALPEL, Ethicon Endo-Surgery, Inc, Cincinnati, Ohio) to create entry holes for the linear stapler in both the duodenum and the remnant stomach; (2) suction and preparation of the first stapler, which started from the insertion of the suction device to suck out the gastric contents until the first stapler was introduced into the abdomen; (3) creation of the common channel, which started from insertion of the first stapler to make a common channel between the duodenum and the remnant stomach, (4) preparation of the second stapler, which comprised the time between when the first stapler was removed from the abdomen and when the second stapler was introduced, and (5) closure of the entry hole, which began with the insertion of the second stapler for closure of the entry hole for the stapler, followed by preparation of a third stapler, and ended with the complete closure of the entry hole using the third stapler (“Supplementary video”). We compared four of these steps among the three groups, excluding the second step. Time for preparation of the stapler was checked to determine differences in preparation of the staplers, because, unlike laparoscopic endolinear staplers, the robotic stapler in this study requires extra time for insertion, loading, and calibration before introducing it into the abdomen and for adjusting the stapler according to tissue thickness. We measured the time for preparing the second stapler because there were fewer extra or distracted movements at this part.

### Statistical analysis

Categorical variables among clinicopathologic characteristics were analyzed using the chi-square test or Fisher's exact test. For continuous variables, the Kruskal–Wallis test was conducted to identify differences among the three groups. The Mann–Whitney U-test was used for post-hoc analysis and was adjusted with the Bonferroni method. All analyses were conducted using SAS version 9.4 (SAS Institute, Cary, NC, USA) and R package version 3.5.1 (The R Foundation for Statistical Computing, Vienna, Austria). All *P* values < 0.05 were considered statistically significant.

## Supplementary information

Supplementary Information.

Supplementary Video.

## Data Availability

The datasets generated during and/or analyzed during the current study are available from the corresponding author on reasonable request.
