# Supplementary material for: Delta-shaped gastroduodenostomy using a robotic stapler in reduced-port totally robotic gastrectomy: its safety and efficiency compared with conventional anastomosis techniques
Source: Sci Rep. 2020 Sep 7;10:14729. doi: 10.1038/s41598-020-71807-z (PMC7477196; doi:10.1038/s41598-020-71807-z)
Supplement: Supplementary file 1 — Supplementary Information. [file 41598_2020_71807_MOESM1_ESM.docx]

**Delta-shaped gastroduodenostomy using a robotic stapler in reduced-port totally robotic gastrectomy: Its safety and efficiency compared with conventional anastomosis techniques**

Ji Su Kim^1^, Hemant Batajoo^1,2^, Taeil Son^1,3^*, Seohee Choi^1,3^, Won Jun Seo^4^, Minah Cho^1,3^, Yoo Min Kim^1,3^, Joong Ho Lee^1,5^, Hyoung-Il Kim^1,3^, Woo Jin Hyung^1, 3^

^1^Department of Surgery, Yonsei University College of Medicine, Seoul, South Korea; ^2^Department of Surgery, Nepal Cancer Hospital & Research Center, Lalitpur, Nepal; ^3^Gastric Cancer Center, Yonsei Cancer Hospital, Yonsei University Health System, Seoul, South Korea; ^4^Department of Surgery, Korea University College of Medicine, Seoul, Korea; ^5^Department of Surgery, Yongin Severance Hospital, Yonsei University College of Medicine, Gyeongi, Korea

**Supplementary video.** Delta-shaped Billroth I anastomosis using robotic stapler

This unedited video illustrates intracoporeal delta-shaped gastroduodesnostomy (Billroth I) technique using DA VINCI robotic ENDOWRIST stapler after reduced-port robotic distal subtotal gastrectomy. To compare the reconstruction time with gastroduodenostomy using laparoscopic endolinear stapler, we divided reconstruction time into five steps as described. The procedure was performed by Dr. T. Son and assisted by a bedside assistant surgeon (Dr. W.J. Seo). The recorded conversation behind the narration was between the surgeon in the console and the bedside assistant surgeon.
